# Beyond antiparasitic activity: elucidating the antibacterial potency of pyrvinium pamoate

**DOI:** 10.1128/spectrum.02158-25

**Published:** 2025-09-30

**Authors:** Angela Alcaraz-Martínez, Paloma Muñoz-Báez, Pablo Peñalver, Juan Carlos Morales, Rubén Cebrián

**Affiliations:** 1 Departamento de Microbiología Clínica, Instituto de Investigación Biosanitaria ibs. GRANADA, Hospital Universitario San Cecilio 616465, Granada, Spain; 2 CIBER de Enfermedades Infecciosas, CIBERINFEC, ISCIII, Madrid, Spain; 3 Departamento de Bioquímica y Farmacología Molecular, Instituto de Parasitología y Biomedicina López Neyra, CSIC 38800, Granada, Spain; 4 Departamento de Fisicoquímica, Facultad de Farmacia, Universidad de Granada, Campus Universitario de Cartuja 16741, Granada, Spain; Università Roma Tre, Roma, Italy

**Keywords:** pyrvinium pamoate, antimicrobial resistance, drug repurposing

## Abstract

Antimicrobial resistance represents a critical global health threat, demanding innovative therapeutic strategies. In this study, we investigate the repurposing potential of pyrvinium pamoate (PP)—a long-established anthelmintic agent—for antibacterial applications. Comprehensive *in vitro* analyses revealed that while gram-negative bacteria exhibited inherent resistance due to limited drug uptake, gram-positive pathogens, particularly within the orders *Actinomycetales* and *Bacillales*, were markedly susceptible at low micromolar concentrations. Enhanced antibacterial efficacy was observed when PP was combined with outer membrane-permeabilizing agents, such as the peptide D11 or pentamidine, which facilitated increased intracellular accumulation. Additionally, the role of efflux pump activity was explored; its inhibition in *Staphylococcus aureus* resulted in significant drug retention and a concomitant reduction in minimum inhibitory concentrations, while disruption of the proton motive force attenuated uptake. The compound demonstrated bactericidal effects against *S. aureus* and a bacteriostatic profile against *Pseudomonas aeruginosa* when sensitized with outer membrane permeabilizing agents. Furthermore, synergistic studies with several antibiotics revealed the potential of PP as a valuable addition to the antimicrobial arsenal against multidrug-resistant pathogens. These findings motivate further mechanistic studies and clinical evaluation of PP in antimicrobial therapy. PP shows promise as a repurposed antibacterial agent, particularly against gram-positive pathogens, with enhanced activity against gram-negative pathogens when combined with membrane-permeabilizing agents or in the presence of efflux pump inhibitors.

**IMPORTANCE:**

Antimicrobial resistance is a growing global crisis that threatens the effectiveness of current treatments. Developing new antibiotics is challenging and time-consuming, so repurposing existing drugs offers a faster alternative. Pyrvinium pamoate (PP) is a well-known antiparasitic drug that has also been studied for cancer treatment, but its antibacterial potential has received little attention. In this study, we show that PP is effective in killing several gram-positive bacteria, including *Staphylococcus aureus*, at low doses. Although gram-negative bacteria are more resistant, we found that combining PP with agents that open up bacterial membranes makes these bacteria more vulnerable. Our research also explains how bacteria take in and remove PP, which can affect how well it works. These findings support the idea of repurposing PP as an antibiotic, especially in combination therapies, to help combat multidrug-resistant infections.

## INTRODUCTION

Antimicrobial resistance (AMR) has emerged as one of the foremost global public health challenges, undermining the effectiveness of existing therapies and contributing to prolonged illnesses, escalating healthcare costs, and increased mortality rates (1). Despite urgent calls to address AMR, the development of new antimicrobial agents has slowed considerably over the past few decades, hindered by scientific, regulatory, and economic barriers (2, 3). Consequently, innovative strategies—ranging from the discovery of novel compounds to the repurposing of existing drugs—are essential to rejuvenate the antibacterial pipeline and combat multidrug-resistant pathogens (4, 5).

Pyrvinium pamoate (PP) is a well-established anthelmintic agent historically used to treat pinworm (*Enterobius vermicularis*) infections in humans (6). Structurally, it is characterized by a cyanine-like core that grants the molecule distinct physicochemical properties, including poor systemic absorption and pronounced tissue-binding capacity (7). Clinically, PP’s efficacy against pinworms has been attributed to its ability to disrupt key metabolic processes within the parasite. In particular, it is believed to interfere with the parasite’s energy metabolism by inhibiting glucose uptake and potentially altering mitochondrial membrane potentials, which ultimately leads to reduced viability of the worm (8). Besides its antihelminthic activity, during the last years, PP has also garnered considerable interest in oncology research, primarily due to its capacity to inhibit the Wnt/β-catenin signaling pathway—a key driver of cancer cell proliferation and survival in various tumor types (9–11). Preclinical studies have demonstrated that PP can suppress the growth of pancreatic, colorectal, breast, and prostate cancer cells (among others), often with minimal effects on non-cancerous cells by binding G-quadruplex DNA structures (12–15). In addition to targeting Wnt signaling, PP has been shown to exert cytotoxic effects through alterations in mitochondrial function and energy metabolism, apparently imitating its parasiticidal properties (16). These findings underscore its potential as a repurposed anticancer agent, with ongoing investigations focused on elucidating its precise molecular targets and optimizing drug formulations to improve efficacy and selectivity in cancer therapy.

Beyond its recognized anthelmintic and anticancer activity, PP has drawn attention in recent years for its potential antimicrobial properties (17–19). Although still under investigation, preliminary findings suggest that it may exhibit activity against certain bacteria, making it a candidate for repurposing as an antibacterial agent. The mechanisms by which PP exerts antibacterial effects have not been conclusively defined, but similarities with its antiparasitic mode of action are possible.

Our study highlights the potential of PP as an antibacterial agent with selective activity against gram-positive bacteria, particularly those within the *Actinomycetales* and *Bacillales* orders. This study expands upon this prior knowledge by exploring PP’s antimicrobial spectrum, uptake mechanisms, and potential resistance determinants.

## RESULTS

### Antimicrobial profile of PP

**Fig 1:**
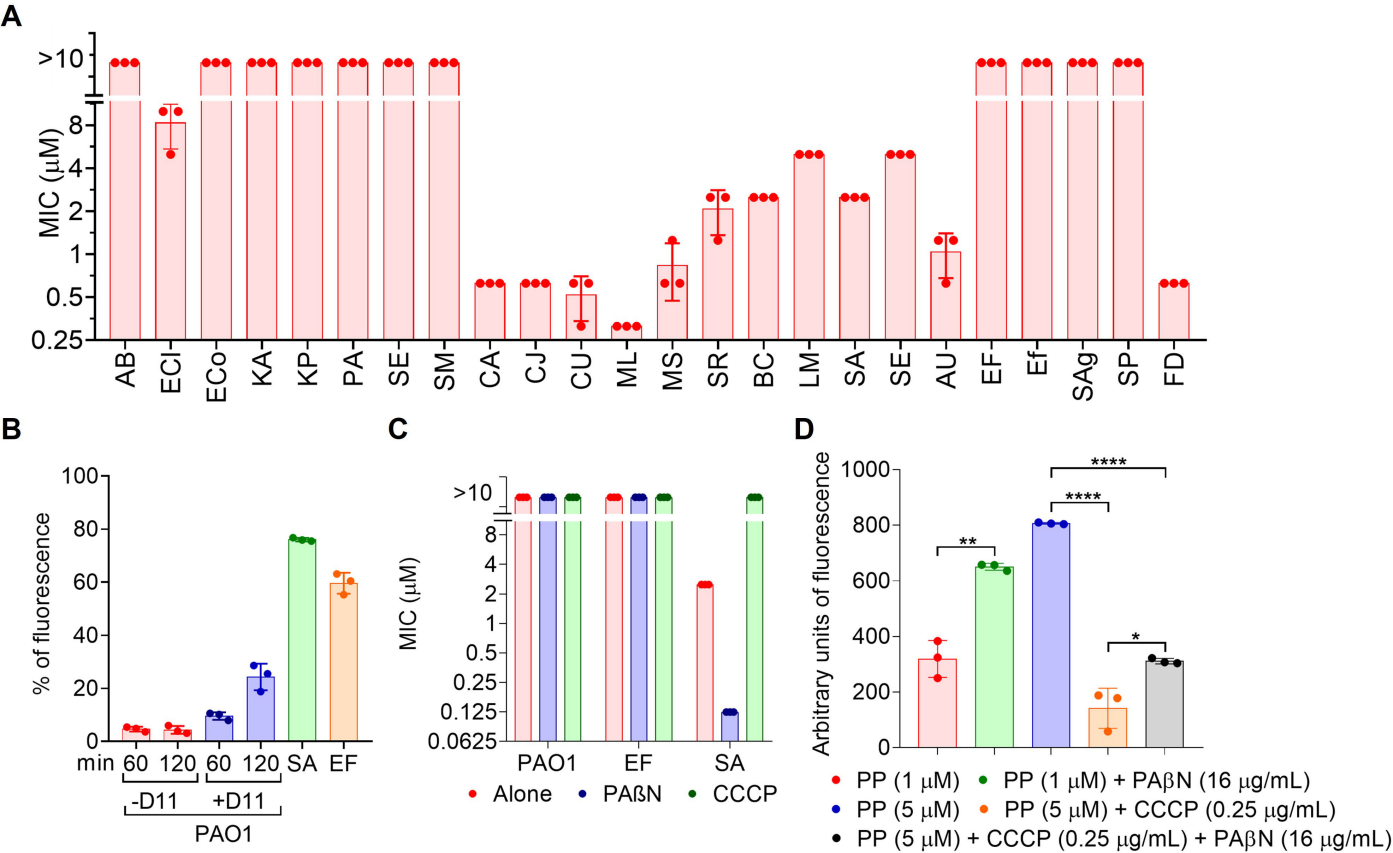
(**A**) Antimicrobial activity of PP against a pathogen collection. (**B**) PP uptake for PAO1 (in the presence or absence of D11 peptide after 60 or 120 min), *S. aureus* HUSC 263091, and *E. faecalis* HUSC 123095 (after 15 min). (**C**) MIC for PP in PAO1, *E. faecalis* HUSC 123095*,* and *S. aureus* HUSC 263091 in the presence or absence of PAβN or CCCP. (**D**) Effect on PP uptake in *S. aureus* in the presence of PAβN or CCCP. * *P* <0.05, ** P <0.01, **** *P* <0.0001.

Initially, we evaluated the antimicrobial profile of PP against a panel of gram-negative (*n* = 8) and gram-positive (*n* = 16) bacteria. As shown in Fig. 1A (Table S1), all gram-negative strains were resistant at the highest concentration tested (10 µM), with *Enterobacter cloacae* exhibiting the lowest minimum inhibitory concentration (MIC) (8.33 µM, Fig. 1A; Table S1). In contrast, PP displayed good efficacy against gram-positive species, most notably those within the order *Actinomycetales* (MIC values ranging from 0.26 µM for *C. urealiticum* to 1.3 µM for *S. radingae*). This was followed by *Bacillales* (MIC values ranging from 2.5 µM for *B. cereus* or *S. aureus* to 5 µM for *S. epidermidis* or *L. monocytogenes*). Members of the *Lactobacillales* were generally as resistant as gram-negative bacteria, except for *Fadklamia* spp., which was the only susceptible strain in that group (MIC 0.625 µM, Fig. 1A; Table S1).

### PP requires drug uptake across the bacterial membranes to be effective

We employed flow cytometry to assess PP uptake in PAO1. As shown in Fig. 1B, no substantial accumulation of PP was observed in PAO1, even after 2 h of exposure, suggesting limited or no entry. In contrast, in the gram-positive bacterium *S. aureus*, approximately 80% of the cells were stained within 15 min of PP administration, indicating that PP readily penetrates these cells. To further investigate the influence of the outer membrane on PP activity, we evaluated its antimicrobial effect against several gram-negative species in the presence of four outer membrane-perturbing agents: the peptide D11 (20), EDTA, polymyxin B (21), and pentamidine (22) (Table 1). First, we determined the MIC of each agent individually (Table S2). We then measured the MIC of PP in combination with sub-MIC concentrations of these permeabilizers (Table S2).

**TABLE 1:** Bacterial strains used in this work

	Strain	Short name	Origin
Gram-positive	*Aerococcus urinae* HUSC 230204	AU	University Hospital San Cecilio (HUSC)
	*Bacillus cereus* HUSC 264402	BC	HUSC
	*Corynebacterium amycolatum* HUSC 256285	CA	HUSC
	*Corynebacterium jeikeium* HUSC 223612	CJ	HUSC
	*Corynebacterium urealiticum* HUSC 235888	CU	HUSC
	*Enterococcus faecalis* HUSC 123095	EF	HUSC
	*Enterococcus faecium* HUSC 263161	Ef	HUSC
	*Fadklamia* sp. HUSC 263425	FD	HUSC
	*Listeria monocytogenes* HUSC 765279	LM	HUSC
	*Micrococcus luteus* UGRA1	ML	University of Granada
	*Mycobacterium smegmatis* UGRA1	MS	University of Granada
	*Schaalia radingae* HUSC 256790	SR	HUSC
	*Streptococcus agalactiae* HUSC 264551	SAg	HUSC
	*Streptococcus pyogenes* HUSC 42856	SP	HUSC
	*Staphylococcus aureus* HUSC 263091	SA	HUSC
	*Staphylococcus epidermidis* HUSC 258042	SE	HUSC
Gram-negative	*Acinetobacter baumannii* ATCC 19606	AB	American Type Culture Collection (ATCC)
	*Enterobacter cloacae* ATCC 13047	ECl	ATCC
	*Escherichia coli* ATCC 25922	ECo	ATCC
	*Klebsiella aerogenes* ATCC 13048	KA	ATCC
	*Klebsiella pneumoniae* ATCC 700603	KP	ATCC
	*Pseudomonas aeruginosa* PAO1	PA	ATCC
	*Salmonella enterica* ATCC 43971	SE	ATCC
	*Stenotrophomonas maltophilia* HUSC 156390	SM	HUSC

As summarized in Table 1, synergistic effects varied according to both the bacterial strain and the outer membrane-permeabilizing agent. Notably, EDTA failed to exhibit synergy with PP in any instance, suggesting that the removal of divalent cations (e.g., Ca²^+^) from the outer membrane does not enhance PP’s activity. Conversely, combining PP with pentamidine significantly reduced its MIC in *A. baumannii*, *E. cloacae*, and *P. aeruginosa*, while polymyxin B produced a similar effect against *Escherichia coli*, *E. cloacae*, and *S. enterica*. Among the outer membrane-permeabilizing agents tested, the peptide D11 proved the most effective, reducing PP’s MIC against all strains, with particularly strong effects observed in *P. aeruginosa* and *S. maltophilia* (Table 1).

To validate these findings, we examined PP uptake via flow cytometry in PAO1, using D11 as the permeabilization agent. Fluorescence was measured at 60 and 120 min to quantify compound internalization. As shown in Fig. 1B, PP uptake was time-dependent and significantly higher in the presence of D11 compared with the control without D11 (Fig. 1B; Table S3). This enhanced intracellular accumulation likely underlies the increased antimicrobial activity observed with the D11–PP combination. However, increased uptake alone does not ensure susceptibility. In *E. faecalis*, which displays high resistance levels to PP (>128 µM), approximately 60% of cells internalized the compound (Fig. 1B; Table S4), yet no antimicrobial effect was detected (Fig. 1A).

### PP is actively pumped inside the cells and removed by efflux pumps

Considering that other gram-positive bacteria such as *Enterococcus* spp. or *Streptococcus* spp. displayed high resistance to PP (Fig. 1A), and the values of PP uptake in *E. faecalis* (Fig. 1B), we explored alternative resistant mechanisms to PP that could be related to such a resistance.

**Fig 2:**
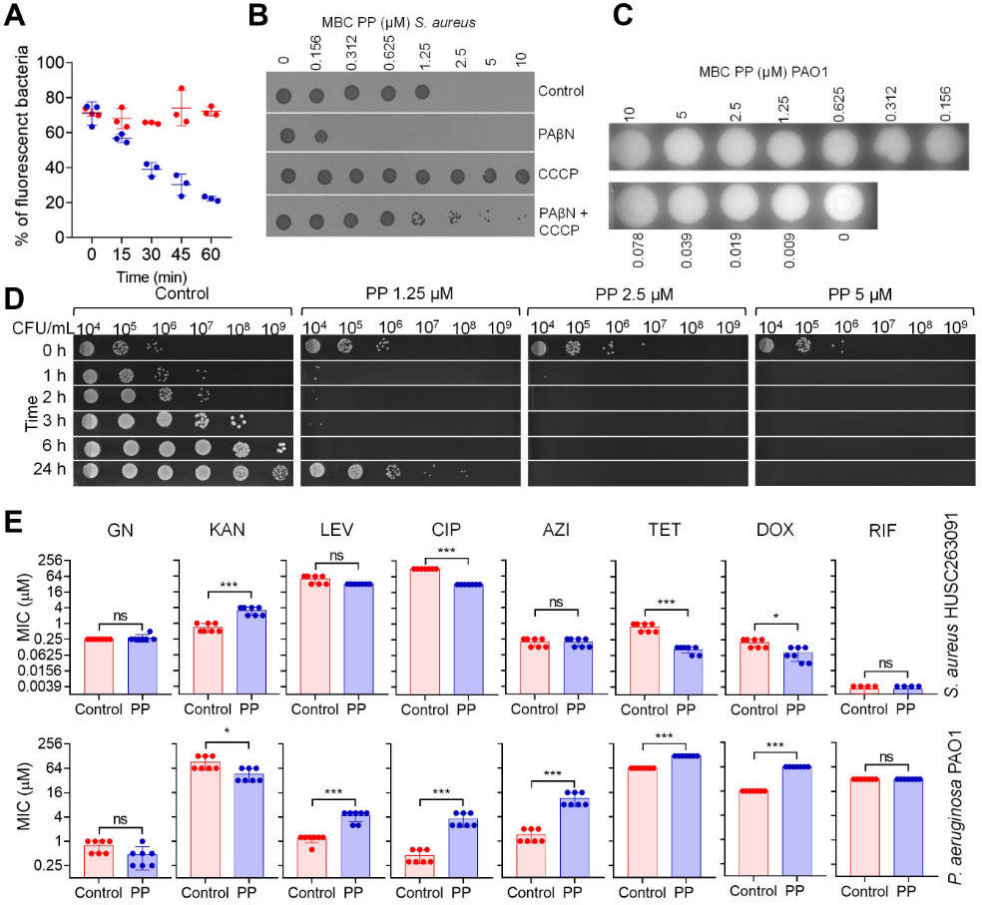
(**A**) Efflux of PP during 1 h in *S. aureus* HUSC 263091 (red) and *E. faecalis* HUSC 123095 (blue). (**B**) MBC for *S. aureus* HUSC 263091 in the presence or absence of PAβN and/or CCCP. (**C**) MBC for PAO1 after D11 sensitization. (**D**) Time-kill assay for PP in *S. aureus* HUSC 263091 for 0.5×, 1×, and 2× the MIC during 24 h. (**E**) Combined activity of PP and gentamycin (GN), kanamycin (KAN), levofloxacin (LEV), ciprofloxacin (CIP), azithromycin (AZI), tetracycline (TET), doxycycline (DOX), and rifampicin (RIF) against *S. aureus* and PAO1. *P <0.05, *** *P* < 0.001

First, we explored the activity of efflux pumps on PP resistance in three strains: *P. aeruginosa*, *E. faecalis,* and *S. aureus*. For that, we used two families of efflux pump inhibitors CCCP and PAβN. Initially, the MIC for each drug was determined for each bacteria and then we performed a MIC for PP at sub-MIC concentrations of these efflux pump inhibitors (Table S4). Interestingly, neither CCCP nor PAβN enhanced the activity of PP in PAO1 or *E. faecalis* strains, suggesting that either the efflux pumps in these bacteria are not the primary resistance mechanism for PP, or other resistance mechanisms overshadow the effects of this efflux pump inhibitor are taking place. However, in *S. aureus,* contrasting results were observed for each inhibitor. While PAβN enhanced the activity of PP, CCCP inhibited its activity. The MIC for PP was reduced from 2.5 to 0.156 µM in the presence of PAβN and increased at least to 10 µM when CCCP was added at sub-MIC concentration. To confirm this effect, we explored the effect of each pump inhibitor on the PP uptake by *S. aureus* by flow cytometry. When PAβN was added, a significant increase in the fluorescence for PP was observed, suggesting that the inhibition of efflux pumps results in a drug accumulation inside the cells (Fig. 1D; Table S5) that could be related to the lower MIC observed when added. However, when CCCP was added, fluorescent was strongly reduced, suggesting that PP uptake is dependent on an intact membrane PMF (Fig. 1D; Table S5). In fact, a similar effect was observed when another PMF disruptor was added (FCCP), the MIC for PP was also increased (5 µM). When we tested other efflux pump inhibitors such as verapamil or reserpine with verified activity against *S. aureus* (23) and *E. faecalis* (24), no activity was observed for *E. faecalis*, and neither or just modest antimicrobial activity (1.25 µM for verapamil) was observed for *S. aureus* (Table S4). Considering these findings, we assessed PP efflux in *S. aureus* and *E. faecalis* using flow cytometry. As depicted in Fig. 2A (Table S6), PP was retained in *S. aureus*, whereas it was actively expelled from *E. faecalis*. These results suggest that *E. faecalis* harbors an efflux system unresponsive to PAβN, reserpine, or verapamil, while *S. aureus* appears to rely on a more limited, PAβN-sensitive efflux mechanism.

### PP is bactericidal for *S. aureus* and bacteriostatic for *P. aeruginosa*

Next, we investigated the biocidal activity of PP against *S. aureus* and PAO1 to determine whether its effect was bactericidal or bacteriostatic. To this end, we performed a standard MIC assay, followed by subculturing 10 µL from the first well showing no visible growth in the fresh culture medium. For *S. aureus*, no differences were observed between the MIC and the minimum bactericidal concentration (MBC), indicating that these compounds exhibit bactericidal activity (Fig. 2B). Notably, the MBC was reduced in the presence of PAβN and abolished by CCCP (Fig. 2B). For PAO1, we assessed the MBC following MIC determination in the presence of D11. Despite an MIC of <0.01 µM, the MBC exceeded 10 µM (Fig. 2C), suggesting that in this gram-negative bacterium, the synergistic effect is bacteriostatic rather than bactericidal. We also evaluated the dead kinetics in the case of *S. aureus*. For that, we tested 0.5× MIC, MIC, and 2× MIC concentrations of PP in MHB inoculated with 10^6^ CFU/mL. As depicted in Fig. 2D, just after 1 h of treatment with PP, almost all bacteria disappear from the plate. After 2 or 3 h of treatment, some colonies were detected in the 0.5× MIC. At 6 h no colonies were detected (<1,000 CFU/mL). However, after 24 h, the cells were recovered at 0.5x MIC, but not at the MIC (Fig. 2D).

### Combined activity with antibiotics

Synergy with conventional antibiotics can enhance overall efficacy, enabling lower doses and fewer side effects. By combining complementary mechanisms, it may also help curb resistance and prolong the usefulness of both new and established antimicrobials. Accordingly, we tested the combined activity of PP with eight antibiotics from different classes—gentamicin (GEN), kanamycin (KAN), levofloxacin (LEV), ciprofloxacin (CIP), azithromycin (AZI), tetracycline (TET), doxycycline (DOX), and rifampicin (RIF)—against *S. aureus* and *P. aeruginosa*.

In an initial fixed-concentration screen (0.25× MIC PP for *S. aureus*; 10 µM PP for *P. aeruginosa* PAO1), antibiotic MIC shifts were modest overall in *S. aureus*, whereas several combinations in *P. aeruginosa* were antagonistic (Fig. 2E). To comprehensively assess interactions, we performed checkerboard assays across a range of PP concentrations and calculated fractional inhibitory concentration indices (FICI). In *S. aureus*, synergy was observed with TET and CIP (Fig. 2E; Fig. S1), and a significant MIC reduction was also seen with DOX (Fig. 2E). Additive effects were noted for some combinations with LEV, AZI, and RIF, while the combination with KAN was antagonistic (Fig. 2E; Fig. S1). In contrast, in the gram-negative strain *P. aeruginosa* PAO1, PP antagonized the antibiotics that were synergistic in *S. aureus*—including AZI—whereas synergy was observed with KAN (Fig. 2E; Fig. S1). These findings suggest that, in *P. aeruginosa*, PP may interfere with antibiotic uptake or entry.

## DISCUSSION

Despite its long-standing use as an anthelmintic, PP has received limited attention as an antimicrobial agent. Only a handful of studies have explored its activity against bacterial pathogens, primarily focusing on gram-positive species such as *S. aureus* or *M. tuberculosis* (17–19, 25, 26) or the intracellular pathogen *Bartonella henselae* (27). This scarcity of data underscores the need for further investigation into its antibacterial potential and mechanisms of action across a broader spectrum of pathogens.

Our findings extend the known antimicrobial profile of PP, suggesting a degree of selectivity toward Actinomycetales, especially *Corynebacterium* spp., followed by Bacillales, while members of the Lactobacillales group and gram-negative bacteria remain resistant. The sensitive bacteria displayed MIC values below the range of PP concentration accepted for oral administration (≈11 mg/kg up to 1 g per dose/day for 2 weeks) and within the range of intraperitoneal dosages (0.1–1 mg/kg daily) currently under investigation for cancer treatments (11, 28).

Intrigued by the lack of activity against gram-negative bacteria, we assessed whether PP could traverse the outer membrane. Using its inherent fluorescence, we examined uptake in *P. aeruginosa* PAO1 and *S. aureus*. PP was unable to enter PAO1 but successfully entered *S. aureus*, indicating that the outer membrane functions as a permeability barrier, as described for many antibiotics (20, 29). When outer membrane perturbing agents were added, gram-negative bacteria became sensitized to PP, especially PAO1. However, unlike the bactericidal effect seen in *S. aureus*, the effect was bacteriostatic.

Pentamidine and polymyxin, which interact with negatively charged lipopolysaccharides in the gram-negative outer membrane by displacing divalent cations such as Mg²^+^ and Ca²^+^, compromise membrane integrity and increase permeability, thus improving antimicrobial uptake (21, 22). Although previous studies show these agents sensitize bacteria to various antibiotics (22, 30), their synergy with PP was not universal; it was both strain- and drug-dependent. In contrast, the peptide D11 sensitized all tested bacteria to PP, as previously described for other pathogens and antibiotics (20). The pronounced efficacy of PP against gram-positive bacteria likely stems from differences in cell envelope architecture. Gram-negative outer membranes limit intracellular accumulation of PP, while their absence in gram-positive bacteria may facilitate greater uptake and retention. This has been observed for other hydrophobic drugs with limited gram-negative activity, including rifamycins and lipophilic cationic peptides (29, 31). Our experiments with membrane-permeabilizing agents confirmed that disrupting the outer membrane significantly increases PP susceptibility in gram-negative strains. This highlights the potential for PP in combination therapies with agents that disrupt membrane integrity or inhibit efflux systems. Similar synergistic effects have been noted with polymyxins combined with intracellular-targeting antimicrobials (32).

Monitoring PP uptake in PAO1 in the presence of D11 over time revealed a gradual intracellular increase, reaching about 20% fluorescence after 2 h, compared to faster uptake in *S. aureus*. Interestingly, resistant gram-positive strains like *E. faecalis* showed high uptake (~60%) without affecting viability, suggesting alternative resistance mechanisms may counteract PP’s activity in *E. faecalis*, whereas in PAO1, more critical targets could be reached. PP has recently been described as a G-quadruplex ligand drug. Therefore, the target’s genomic location and the essentiality of the gene(s) involved could influence activity (11). Additionally, efflux pumps play a crucial role in resistance by expelling drugs from bacterial cells (33). To explore their role in PP resistance, we tested two efflux pump inhibitors: CCCP and PAβN. CCCP disrupts the proton motive force (PMF) across the membrane, affecting both Δψ and ΔpH components (34, 35), which are essential for efflux pump function (36). PAβN, in contrast, directly binds to efflux pumps, blocking their activity and increasing intracellular drug concentration (37). While neither inhibitor affected PAO1 or *E. faecalis*, in *S. aureus*, the MIC for PP decreased with PAβN and increased with CCCP. This suggests that PAβN-inhibited pumps may mediate PP resistance in *S. aureus*, and that membrane potential is required for PP activity.

Though PAβN is best known for inhibiting RND efflux pumps in gram-negative bacteria (38), which are scarcely reported in *S. aureus* (39–41), similar effects have been observed with combinations of PAβN and bile salts, enhancing drug activity where reserpine did not (42). PAβN also increased the efficacy of benzalkonium and flavonol derivatives in *S. aureus* (43, 44). *S. aureus* primarily relies on MFS, SMR, MATE, and ABC transporters (41), none of which are robustly inhibited by PAβN as in gram-negatives, but off-target or indirect effects cannot be excluded.

Finally, we explored PP’s potential synergy with a range of antibiotics. While combinations with PP in PAO1 were mostly antagonistic, KAN displayed a synergistic effect. In *S. aureus*, a significant reduction in the MIC was observed with CIP, TET, and DOX, while KAN was antagonistic. Synergistic effects with quinolones (CIP) were previously reported for *S. aureus* (18, 25). However, the observed antagonism with gram-negative strains and synergy with tetracyclines in *S. aureus* of KAN in PAO1 are novel findings.

From a translational perspective, the administration, distribution, metabolism, excretion, and toxicity profile of PP warrants brief comment. As the pamoate salt, PP exhibits negligible systemic absorption after oral dosing; in a classic volunteer study, 350 mg single doses yielded no detectable drug in blood or urine up to 4 days, and ~90% of the dose was recovered in feces (45, 46). Consequently, systemic distribution after oral administration is expected to be minimal. When systemic exposure is achieved (e.g., via alternative formulations, salts, or routes), PP behaves as a lipophilic cation with preferential mitochondrial localization, a property that may influence tissue distribution and off-target effects (7). Clinically, historical use as an anthelmintic indicates a generally favorable tolerability profile dominated by gastrointestinal effects and benign red discoloration of stools/vomit; however, formal characterization of safety and pharmacokinetics for systemic repurposing is ongoing in a Phase I oncology study (47). These features contextualize our MIC findings and support cautious interpretation of systemic antibacterial applications of PP while encouraging formulation and delivery strategies tailored to the intended site of action.

In conclusion, the growing crisis of AMR needs the development of novel therapeutic agents and innovative treatment strategies. Drug repurposing is an attractive approach to accelerate the discovery of new antimicrobial agents, circumventing many of the financial and regulatory difficulties associated with *de novo* drug development. Our study provides compelling evidence for PP’s antibacterial potential, particularly against gram-positive pathogens. By further elucidating its mechanism of action and resistance determinants, PP could serve as a foundation for the development of novel antimicrobial therapies or combination regimens targeting multidrug-resistant bacteria. The appeal of PP for antimicrobial drug repurposing is further supported by its advantageous drug profile, including extensive clinical experience with a relatively favorable safety record and well-defined pharmacological properties. As new antimicrobials are critically needed to address the growing threat of multidrug-resistant pathogens, exploring established drugs like PP provides a timely and cost-effective avenue for discovering novel antibacterial therapeutics. By elucidating its precise molecular targets and optimizing its formulation or delivery, PP could become a valuable addition to the declining arsenal of antimicrobial agents.

## MATERIALS AND METHODS

### Bacterial strains and reagents

PP was tested against a range of pathogenic bacteria, both gram-negative and gram-positive from clinical collection as well as clinically isolated (Table 2).

**TABLE 2:** Combined antimicrobial activity of various outer membrane-permeabilizing agents and PYR against gram-negative bacteria^*a*^

	MIC for PP (µM)			
Strain	D11	EDTA	Pent	Poly B
*A. baumannii* ATCC 19606	1.25	>10	1.04 ± 0.36	>10
*E. cloacae* ATCC 13047	0.41 ± 0.18	5	0.52 ± 0.18	0.31
*E. coli* ATCC 25922	1.25	>10	>10	8.3 ± 2.9
*K. aerogenes* ATCC 13048	2.5	>10	>10	>10
*P. aeruginosa* PAO1	<0.01	10	1.25	>10
*S. enterica* ATCC 43971	8.3 ± 2.9	>10	>10	8.3 ± 2.9
*S. maltofilia* HUSC156390	0.83 ± 0.36	>10	>10	>10

^*a*^ Pent, pentamidine; Poly B, polymyxin B.

Bacterial strains were cultivated in cation-adjusted Mueller-Hinton Broth (Thermo Scientific), supplemented with 5% lysed horse blood when required, at 37°C with continuous aeration. For solid media preparations, 1.5% agarose was added as needed (MHA). PP was purchased from TargetMol, while CCCP and FCCP were obtained from Thermo Scientific. Phe-Arg β-naphthylamide dihydrochloride (PAβN), reserpine, verapamil, and all tested antibiotics were sourced from Sigma-Aldrich.

### MIC, MBC, time-killing test, and combined test

MIC was determined according to the Clinical and Laboratory Standards Institute guideline (48). Briefly, twofold serial dilutions of the antimicrobials were prepared in cation-adjusted MHB in a 96-well plate. The plate was then inoculated with a bacterial suspension standardized to a 0.5 McFarland turbidity (approximately 1.5 × 10^8^ CFU/mL) and diluted to a final concentration of 5 × 10^5^ CFU/mL. When required, 5% lysed horse blood was added. Plates were incubated at 37°C for 24 h, and the MIC was defined as the lowest concentration of the antibiotic that completely inhibited visible bacterial growth. For the MBC assay, after performing the MIC test, 10 µL of culture medium was removed from wells with no visible growth and plated on MHA. In the case of *Pseudomonas aeruginosa*, the MBC was determined following a MIC test for PP conducted in the presence of 4 µM of the D-11 outer membrane-disrupting peptide. The MBC was defined as the lowest concentration at which no visible growth was observed on the agar plate. For the PP MIC determination in the presence of CCCP, FCCP, PAβN, verapamil, reserpine, and D11, the MIC for these drugs was initially determined, and after that, an MIC for PP was performed in the presence of 0.25× their MIC. Time-kill assays were performed to determine the bactericidal or bacteriostatic activity of PP. *S. aureus* culture was adjusted to approximately 1 × 10^6^ CFU/mL in MHB and exposed to PP at 0.5× MIC, 1× MIC, and 2× MIC concentrations. Aliquots were taken at 0, 1, 2, 3, 6, and 24 h, serially diluted 10-fold, and plated onto MHB agar to quantify viable bacteria. Bactericidal activity was defined as a ≥3 log_10_ reduction in CFU/mL relative to the initial inoculum.

In the case of the antibiotics, the MIC for them was determined for *Staphylococcus aureus* in the presence and absence of 0.5×, 0.25×, 0.125×, and 0.0625× MIC of PP (1.25, 0.625, 0.312, and 0.156 µM), while for PAO1, 5, 10, 15, and 20 µM of PP was added due to its inherent resistance. In the case of azithromycin, bismuth subcitrate (TCI Europa) at 8 µM was added to sensitize PAO1 to this antimicrobial. The Fractional Inhibitory Concentration Index (FICI) was calculated as FICI = FIC_A_ + FI_CB_, where FIC_A_ = MIC_A_ (MIC antibiotic in combination)/MIC_A_ (MIC antibiotic alone) and FIC_B_ = MIC_B_(MIC PP in combination)/MIC_B_ (MIC PP alone). Interpretation followed European Committee on Antimicrobial Susceptibility Testing criteria: synergistic, FICI ≤ 0.5; additive, 0.5 < FICI ≤ 1; indifferent, 1 < FICI ≤ 2; and antagonistic, and FICI > 2. When the MIC of an agent was not reached within the tested concentration range, a value equal to two times the highest concentration tested was used for the calculation.

### Outer membrane permeabilization test

To investigate the role of the outer membrane in PP uptake, we determined the MIC of PP in the presence of four outer membrane-disrupting agents: EDTA, pentamidine, polymyxin B, and the peptide D-11. Initially, the MIC for each agent was established individually. Subsequently, PP’s MIC was determined in the presence of sub-MIC concentrations of these compounds against seven different gram-negative bacterial strains. The concentrations used for the combinatorial tests are listed in Table S2.

### Evaluation of PP uptake and PP efflux by flow cytometry

Taking advantage of the fluorescent properties of PP, we evaluated its uptake in *S. aureus*, *E. faecalis*, and *P. aeruginosa*. To prepare the cells, a fresh MHB culture was centrifuged, washed in G-HEPES buffer (HEPES supplemented with 5 mM glucose, pH 7.4), and adjusted to a cell concentration of 0.5 McFarland turbidity (about 1.5 × 10^8^ CFU/mL) in the same buffer. For SA and EF, the prepared cells were incubated with 1 µM PP for 15 min, after which they were analyzed using a FACSymphony A3 (Becton Dickinson) flow cytometer with excitation and emission set at 561 and 586 nm, respectively. For PAO1, the uptake was assessed in the presence and absence of the D-11 peptide (4 µM) following 60 and 120 min of PP exposure.

To evaluate PP efflux, SA and EF cells (adjusted to a 0.5 McFarland standard) were incubated with 1 µM PP in G-HEPES buffer for 15 min. The cells were then washed three times (to remove PP) and resuspended in the same buffer, and fluorescence was recorded by flow cytometry every 15 min for 1 h. An analogous assay was performed for SA in the presence of PAβN, CCCP, or a combination of both. For PAβN, the cells were incubated with 1 µM PP in the presence or absence of 16 µg/mL PAβN, and fluorescence accumulation was evaluated after 15 min. For CCCP, the cells were treated with 5 µM PP in the presence or absence of 0.25 µg/mL CCCP, and fluorescence was similarly monitored. All flow cytometry data were analyzed using the free software Floreada.io (https://floreada.io/).

### Statistical analysis and reproducibility

All experiments were conducted in replicates as described, and the data were analyzed using GraphPad Prism 8 software. Statistical comparisons between the two groups were performed using an unpaired Student’s *t*-test where appropriate.

## SUPPLEMENTAL MATERIAL

Fig. S1 spectrum.02158-25-s0001.tiff FICI calculations for the different concentrations of PP tested in the combinatorial test with antibiotics.

Supplemental material spectrum.02158-25-s0002.docx Tables S1 to S6; Fig. S1 caption.

ASM does not own the copyrights to Supplemental Material that may be linked to, or accessed through, an article. The authors have granted ASM a non-exclusive, world-wide license to publish the Supplemental Material files. Please contact the corresponding author directly for reuse.
