# Supplementary figures and images for: Beyond antiparasitic activity: elucidating the antibacterial potency of pyrvinium pamoate
Source: Microbiol Spectr. 2025 Sep 30;13(11):e02158-25. doi: 10.1128/spectrum.02158-25 (PMC12584725; doi:10.1128/spectrum.02158-25)

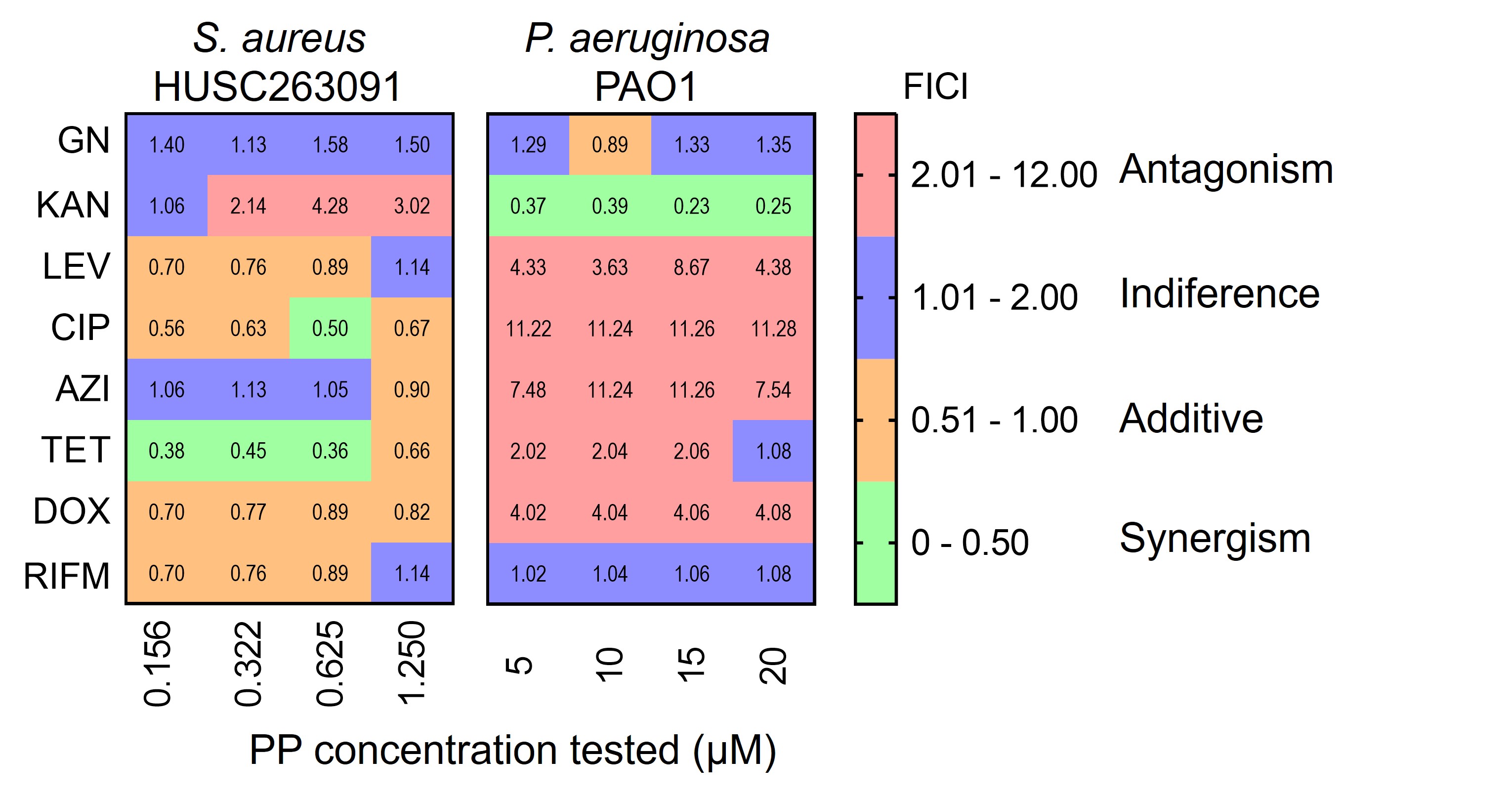

Supplement: Fig. S1 — FICI calculations for the different concentrations of PP tested in the combinatorial test with antibiotics. [file spectrum.02158-25-s0001.tiff]
