# Supplementary material for: Beyond antiparasitic activity: elucidating the antibacterial potency of pyrvinium pamoate
Source: Microbiol Spectr. 2025 Sep 30;13(11):e02158-25. doi: 10.1128/spectrum.02158-25 (PMC12584725; doi:10.1128/spectrum.02158-25)
Supplement: Supplemental material — Tables S1 to S6; Fig. S1 caption. [file spectrum.02158-25-s0002.docx]

**Supplementary Table 1:** Minimal Concentration Inhibition (MIC) for PP for the tested bacterial collection.

|  | **Strain** | **MIC (µM)** |
| --- | --- | --- |
| **Gram-positive** | *Aerococcus urinae* HUSC 230204 | 1.04±0.36 |
|  | *Bacillus cereus* HUSC 264402 | 2.5 |
|  | *Corynebacterium amycolatum* HUSC 256285 | 0.625 |
|  | *Corynebacterium jeikeium* HUSC 223612 | 0.625 |
|  | *Corynebacterium urealiticum* HUSC 235888 | 0.521±0.18 |
|  | *Enterococcus faecalis* HUSC 123095 | >10 |
|  | *Enterococcus faecium* HUSC 263161 | >10 |
|  | *Fadklamia* sp. HUSC 263425 | 0.625 |
|  | *Listeria monocytogenes* HUSC 765279 | 5 |
|  | *Micrococcus luteus* UGRA1 | 0.312 |
|  | *Mycobacterium smegmatis* UGRA1 | 0.833±0.36 |
|  | *Schaalia radingae* HUSC 256790 | 2.08±0.72 |
|  | *Streptococcus agalactiae* HUSC 264551 | >10 |
|  | *Streptococcus pyogenes* HUSC 42856 | >10 |
|  | *Staphylococcus aureus* HUSC 263091 | 2.5 |
|  | *Staphylococcus epidermidis* HUSC 258042 | 5 |
| **Gram-negative** | *Acinetobacter baumannii* ATCC 19606 | >10 |
|  | *Enterobacter cloacae* ATCC 13047 | 8.33±2.88 |
|  | *Escherichia coli* ATCC 25922 | >10 |
|  | *Klebsiella aerogenes* ATCC 13048 | >10 |
|  | *Klebsiella pneumoniae* ATCC 700603 | >10 |
|  | *Pseudomonas aeruginosa* PAO1 | >10 |
|  | *Salmonella enterica* ATCC 43971 | >10 |
|  | *Stenotrophomonas maltophilia* HUSC 156390 | >10 |

**Supplementary Table 2:** MIC for the outer membrane permeabilizing agents tested and concentration “Used” for the combined activity with pyrvinium pamoate.

|  | **Concentration (µM)** | | | | | | | |
| --- | --- | --- | --- | --- | --- | --- | --- | --- |
|  | **D11** | | **EDTA** | | **Pent** | | **Poly B** | |
| **Strain** | MIC | Used | MIC | Used | MIC | Used | MIC | Used |
| *A. baumannii* ATCC 19606 | 4 | 1 | 291.6 | 75 | >420 | 420 | 0.09 | 0.022 |
| *E. cloacae* ATCC 13047 | 16 | 4 | >1000 | 500 | 420 | 105 | 5.7 | 1.44 |
| *E. coli* ATCC 25922 | 4 | 1 | 416.6 | 105 | 210 | 52.5 | 0.36 | 0.09 |
| *K. aerogenes* ATCC 13048 | 32 | 4 | >1000 | 500 | >420 | 420 | 0.14 | 0.037 |
| *P. aeruginosa* PAO1 | >32 | 4 | >1000 | 500 | >420 | 420 | 0.18 | 0.045 |
| *S. enterica* ATCC 43971 | 4 | 1 | >1000 | 500 | 420 | 105 | 0.6 | 0.14 |
| *S. maltofilia* HUSC156390 | >32 | 8 | 375 | 95 | >420 | 420 | 0.09 | 0.022 |

**Supplementary Table 3**: Uptake of PP for PAO1 in the presence and absence of the outer membrane permeabilizing agent D-11, and for *S. aureus* and *E. faecalis*.

|  |  | **% of fluorescence** | | | | | |
| --- | --- | --- | --- | --- | --- | --- | --- |
|  | **Time (min)** | **Test 1** | **Test 2** | **Test 3** | **Average** | | |
| PAO1 -D11 | 60 | 3.67 | 4.93 | 5.42 | 4.7 | ± | 0.9 |
|  | 120 | 6.02 | 3.15 | 3.93 | 4.4 | ± | 1.4 |
| PAO1 +D11 | 60 | 10.2 | 10.6 | 7.96 | 9.6 | ± | 1.4 |
|  | 120 | 28.6 | 18.8 | 25.5 | 24.3 | ± | 5.0 |
| *S. aureus* HUSC 263091 | - | 75.5 | 76.8 | 75.9 | 76.1 | ± | 0.6 |
| *E. faecalis* HUSC 123095 | - | 63.1 | 60.5 | 55.3 | 59.6 | ± | 3.9 |

**Supplementary Table 4**: MIC for the efflux pump inhibitor tested and concentration “Used” for the combined activity with pyrvinium pamoate

|  | **Concentration (µg/mL)** | | | | | | | | | |
| --- | --- | --- | --- | --- | --- | --- | --- | --- | --- | --- |
|  | **CCCP** | | **PAβN** | | **FCCP** | | **Reserpine** | | **Verapamil** | |
| **Strain** | MIC | Used | MIC | Used | MIC | Used | MIC | Used | MIC | Used |
| *P. aeruginosa* PAO1 | >128 | 64 | >128 | 32 | >128 | 64 | - | - | - | - |
| *S. aureus* HUSC 263091 | 1 | 0.25 | 64 | 16 | 0.33 | 0.07 | 6.5 | 1.62 | 12.12 | 3 |
| *E. faecalis* HUSC 123095 | 2.5 | 0.625 | 128 | 64 | 32 | 8 | >78 | 78 | >60 | 60 |

**Supplementary Table 5:** Uptake of PP in the presence and absence of PAβN or CCCP.

|  | **Arbitrary units of fluorescence** | | | | | |
| --- | --- | --- | --- | --- | --- | --- |
|  | **Test 1** | **Test 2** | **Test 3** | **Average** | | |
| PP (1 µM) | 383 | 324 | 250 | 319 | ± | 66.6 |
| PP (1 µM) + PAβN (16 µg/mL) | 657 | 658 | 636 | 650.3 | ± | 12.4 |
| PP (5 µM) | 804 | 806 | 810 | 806.6 | ± | 3.0 |
| PP (5 µM) + CCCP (0.25 µg/mL) | 188 | 178 | 58 | 141.3 | ± | 72.3 |
| PP (5 µM) + CCCP (0.25 µg/mL) + PAβN (16 µg/mL) | 307 | 304 | 322 | 311 | ± | 9.6 |

**Supplementary Table 6**: PP efflux for *S. aureus*  and  *E. faecalis* during 1 h.

|  |  |  |  | |  | |  |  | |  |
| --- | --- | --- | --- | --- | --- | --- | --- | --- | --- | --- |
|  |  | **% of fluorescence** | | | | | | | | |
|  | **Time (min)** | **Test 1** | | **Test 2** | | **Test 3** | **Average** | | | |
| *S. aureus* HUSC 263091 | 0 | 73.86 | | 70.81 | | 69.88 | 71.5 | ± | 2.0 | |
|  | 15 | 74.43 | | 66.28 | | 63.3 | 68.0 | ± | 5.7 | |
|  | 30 | 65.9 | | 64.94 | | 65.86 | 65.5 | ± | 0.5 | |
|  | 45 | 85.38 | | 70.35 | | 66.31 | 74.0 | ± | 10.0 | |
|  | 60 | 71.08 | | 75.14 | | 70.25 | 72.1 | ± | 2.6 | |
| *E. faecalis* HUSC 123095 | 0 | 74.55 | | 63.33 | | 74.98 | 70.9 | ± | 6.6 | |
|  | 15 | 54.41 | | 56.52 | | 59.22 | 56.7 | ± | 2.4 | |
|  | 30 | 34.64 | | 42.09 | | 40.16 | 38.9 | ± | 3.8 | |
|  | 45 | 23.58 | | 35.71 | | 31.21 | 30.1 | ± | 6.1 | |
|  | 60 | 22.3 | | 23.54 | | 20.93 | 22.2 | ± | 1.3 | |

**Supplementary Figure 1**: FICI calculations for the different cocnetrations of PP tested in the combinatorial test with antibiotics.
